# Phase I clinical trial to assess safety and efficacy of Oraxol, a novel oral paclitaxel chemotherapy agent, in patients with previously treated metastatic breast cancer

**DOI:** 10.1002/mco2.70097

**Published:** 2025-02-17

**Authors:** Yunfang Yu, Ying Wang, Luhui Mao, Suiwen Ye, Xiuping Lai, Junyi Chen, Yiwen Zhang, Jieqiong Liu, Junyan Wu, Tao Qin, Herui Yao

**Affiliations:** ^1^ Guangdong Provincial Key Laboratory of Malignant Tumor Epigenetics and Gene Regulation, Guangdong‐Hong Kong Joint Laboratory for RNA Medicine, Phase I Clinical Trial Centre, Department of Medical Oncology Breast Tumor Centre, Sun Yat‐sen Memorial Hospital, Sun Yat‐sen University Guangzhou China; ^2^ Faculty of Medicine Macau University of Science and Technology Taipa China

**Keywords:** efficacy, metastatic breast cancer, oraxol, phase I clinical trial, safety

## Abstract

Oraxol, a novel oral paclitaxel chemotherapy agent, has emerged as a potential alternative for treating metastatic breast cancer (MBC). However, its safety and efficacy remain uncertain due to insufficient evidence supporting it. This open‐label, single‐arm, phase I trial was designed to assess the pharmacokinetics, safety, and preliminary antitumor activity of Oraxol in previously treated MBC. The primary objective was to investigate the pharmacokinetics of Oraxol, while secondary endpoints included assessing safety, tolerability, and antitumor activity. Twenty‐four patients (median age, 53 years) were enrolled, and pharmacokinetic analysis showed consistent and reproducible absorption of Oraxol. Note that 96% patients experienced treatment‐related adverse events (TRAEs) and no deaths attributed to TRAEs. The overall response rate was 34.8%, including 34.8% achieving partial response and 56.5% having stable disease. The median follow‐up was 45.7 months, with median progression‐free survival (PFS) of 3.41 months and median overall survival of 17.80 months. Notably, among patients with triple‐negative breast cancer, the disease control rate was 100%, and the median PFS was 8.90 months, which notably exceeded the outcomes observed in other subtypes. Oraxol significantly alters metabolism and correlates with response and survival. In conclusion, Oraxol exhibited promising antitumor efficacy and manageable safety profiles in MBC patients.

## Graphical abstract

This phase I clinical trial demonstrates the safety, efficacy, and survival of Oraxol. They analyze the correlation between metabolites and Oraxol. The study provides valuable evidence supporting the promising antitumor activity and manageable safety profile of Oraxol in patients with metastatic breast cancer, including those with TNBC.

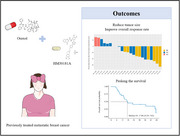

## 1 INTRODUCTION

Breast cancer remains the primary cause of cancer‐related deaths globally and in China. Although early detection, screening, and perioperative treatment have improved cure and survival rates for early‐stage breast cancer,^1^, ^2^, ^3^ the prognosis of metastatic breast cancer (MBC) remains exceedingly poor, with a 5‐year overall survival (OS) rate of less than 20%,^4^, ^5^ and the median survival time for patients with MBC is approximately 2–3 years.^6^ This stark reality underscores the critical need for the development of more effective and personalized therapeutic approaches for MBC. The treatments such as chemotherapy, endocrine therapy, or targeted therapy can improve quality of life and prolong OS.^7^, ^8^ After multiple lines of treatment, MBC becomes refractory to standard therapies, leading to rapid disease progression.^9^ Therefore, it is urgent to develop novel therapeutic strategies that can improve the survival outcomes and quality of life of MBC patients.

Paclitaxel, as a classic microtubule‐stabilizing agent,^10^ is a cornerstone in breast cancer treatment, primarily administered intravenously. The traditional form of paclitaxel, including albumin‐bound paclitaxel, remains effective as monotherapy or in combination for patients with advanced breast cancer across all molecular subtypes, including those resistant to conventional paclitaxel and those who have failed multiple lines of chemotherapy, and early administration is associated with better prognosis.^11^, ^12^ However, the production and clinical preparation of albumin‐bound paclitaxel are complex and expensive, so that patient compliance is often low. Therefore, improving the formulation of paclitaxel to reduce toxicity, lower costs, and enhance patient compliance is an urgent need in the management of MBC.

Liporaxel (RMX3001) has demonstrated efficacy comparable to paclitaxel injection in advanced gastric cancer patients after second‐line treatment, with a lower incidence of adverse effects such as neurotoxicity, leukopenia, and alopecia.^13^, ^14^ However, the lipid self‐emulsifying structure of Liporaxel results in low bioavailability, which significantly restricts the clinical efficacy of Liporaxel. In addition, there is a significant paucity of studies on the pharmacokinetic parameters, dose optimization, and long‐term safety of Liporaxel in MBC.^15^

Oraxol is a new combination of oral paclitaxel capsules and HM30181A methanesulfonate monohydrate. HM30181A inhibits the exocytosis of intestinal P‐glycoprotein (P‐gp), thereby enhancing the absorption and bioavailability of oral paclitaxel capsules.^16^ Other oral paclitaxel has shown promising efficacy and tolerability in treating recurrent or metastatic HER2‐negative breast cancer.^14^, ^17^ However, Oraxol lacks additional evidence from clinical studies to support its safety and efficacy in MBC. Further studies are essential to assess the clinical efficacy and pharmacological properties of Oraxol in MBC.

Hence, this phase I trial aimed to evaluate the pharmacokinetics, safety, and efficacy of Oraxol in patients with MBC. In addition, the study will provide insights into the effects of Oraxol on the metabolic processes of MBC and identify metabolites that could serve as potential biomarkers for efficacy prediction.

## 2 RESULTS

### 2.1 Study participants

**FIGURE 1:**
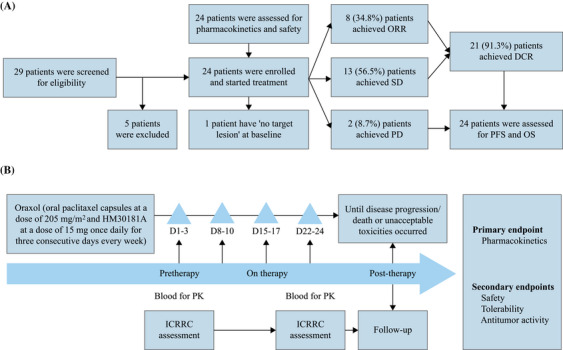
Trial design. (A) Flowchart of patients; (B) trial schema. DCR, disease control rate; ICRRC, Independent Central Radiology Review Committee; ORR, overall response rate; PD, progressive disease; SD, stable disease.

From April 2019 to December 2019, 24 patients were enrolled and treated with Oraxol (Figure 1). Among them, 67% had three or more metastatic sites, and 92% had two or more metastatic sites. The median prior lines of treatment in the metastatic setting were 2 (range 1–5), with 83% having received prior taxane therapy. Detailed patient demographics is presented in Table 1.

**TABLE 1:** Patient demographics and baseline characteristics.

Demographic or characteristic	All patients (*N* = 24)
Age (years)	
Median age, years (range)	53 (35, 70)
Age group, No. (%)	
<65 years	19 (79%)
≥65 years	5 (21%)
ECOG score, No. (%)	
0	0
1	24 (100%)
Subtypes, No. (%)	
Triple negative	4 (17%)
ER(+)/PR(+)	20 (83%)
ER(+)/PR(+) and HER2 positive	2 (8%)
ER(+)/PR(+) and HER2 negative	15 (63%)
Number of metastatic sites, No. (%)	
1	2 (8%)
2	6 (25%)
≥3	16 (67%)
At least one previous chemotherapy	23 (96%)
Number of previous chemotherapy regimens, No. (%)	
0	1 (4%)
1	7 (29%)
2	3 (13%)
≥3	13 (54%)
Prior taxane treatment, No. (%)	20 (83%)
Prior surgery, No. (%)	22 (92%)
At least one previous endocrine therapy, No. (%)	20 (83%)
Treatment lines, No. (%)	
Median (range)	2 (1, 5)
≥3	10 (41.7%)
The median follow‐up time, months (95% CI)	45.7 (95% CI, 42.2–49.2)

Abbreviations: ECOG, Eastern Cooperative Oncology Group; ER, estrogen receptor; HER2, human epidermal growth factor receptor 2; PR, progesterone receptor.

### 2.2 Pharmacokinetics

**FIGURE 2:**
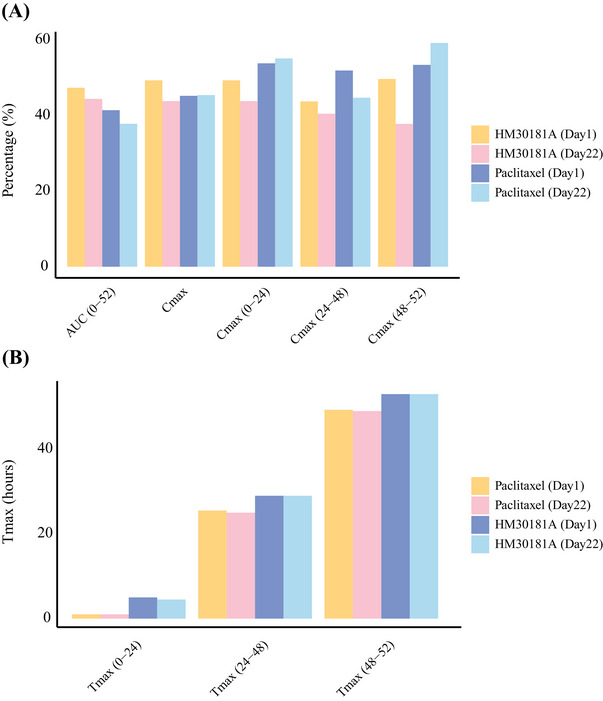
Pharmacokinetic parameters for Oraxel. (A) Consistent and reproducible absorption of Oraxol; (B) pharmacokinetic parameters remained similar between weeks 1 and 4 of treatment.

In the pharmacokinetic analysis, 24 patients received Oraxol treatment for up to 16 weeks, showing consistent and reproducible absorption of Oraxol (Figure 2A). Pharmacokinetic parameters remained similar between weeks 1 and 4 of treatment (Figure 2B). Detailed pharmacokinetic data can be found in Table S1.

### 2.3 Safety

In the safety assessment of 24 patients, 96% experienced treatment‐related adverse events (TRAEs), but no hypersensitivity reactions or TRAE‐related deaths occurred (Table 2). Treatment‐related serious adverse events (SAEs) occurred in 13% of patients, but they were nonfatal. Two patients (8%) withdrew from the study medication due to TEAEs. Among the patients, 63% of patients had grade ≥3 TRAEs, the most common adverse events (AEs) were a decrease in neutrophil count and a reduction in white blood cell count (Table S2). On average, patients received the study treatment for 13 weeks at an average daily dose of 172 mg/m^2^ Oraxol. Some patients required dose reductions due to TRAEs, and dose interruptions were mainly TRAE related.

**TABLE 2:** Summary of treatment‐related adverse event (TRAE) and treatment‐emergent adverse event (TEAE) in all metastatic breast cancer patients.

Preferred TEAE	All patients (*N* = 24)
**Any TEAE, No. (%)**	24 (100%)
TEAE related to Oraxol	23 (96%)
Serious TEAE	3 (13%)
Serious TEAE related to Oraxol	1 (4%)
TEAE leading to dose interruption or reduction	15 (63%)
TEAE leading to study drug discontinuation	2 (8%)
TEAE leading to death	0
**TRAE reported in ≥10% of all patients**	
**Investigations, No. (%)**	22 (92%)
Neutrophil count decreased	20 (83%)
White blood cell count decreased	20 (83%)
Alanine aminotransferase increased	8 (33%)
Aspartate aminotransferase increased	7 (29%)
**Gastrointestinal disorders, No. (%)**	18 (75%)
Abdominal pain	11 (46%)
Diarrhoea	11 (46%)
Nausea	6 (25%)
Abdominal distension	4 (17%)
Vomiting	4 (17%)
**Skin and subcutaneous tissue disorders, No. (%)**	8 (33%)
Alopecia	7 (29%)
**Blood and lymphatic system disorders, No. (%)**	6 (25%)
Anaemia	6 (25%)
**Nervous system disorders, No. (%)**	6 (25%)
Neuropathy peripheral	5 (21%)
**General disorders and administration site conditions, No. (%)**	5 (21%)
Asthenia	4 (17%)
**Metabolism and nutrition disorders, No. (%)**	4 (17%)
Decreased appetite	4 (17%)

### 2.4 Efficacy

**FIGURE 3:**
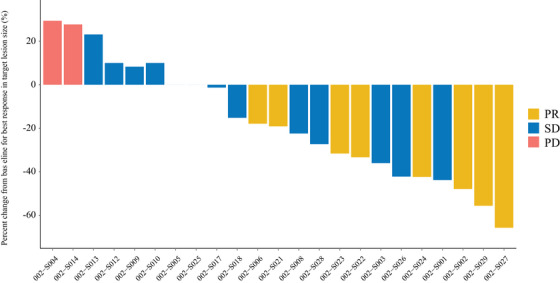
Tumor response assessment with waterfall plot. DCR, disease control rate; ORR, overall response rate; PD, progressive disease; SD, stable disease.

In the full analysis set (FAS), all patients completed at least one complete cycle of Oraxol. The percentage of change from baseline to response in target lesion size for all response‐evaluable patients is depicted in Figure 3. Among the FAS patients (*n* = 23), 34.8% achieved a partial response (PR), resulting in an overall response rate (ORR) of 34.8% (8 out of 23). Furthermore, 56.5% (13 of 23) of patients had stable disease (SD), while 8.7% (2 of 23) demonstrated progressive disease (PD). Patients with prior taxane use had a lower ORR compared to those without (25.0% vs. 45.5%), as did those with prior capecitabine, gemcitabine, or vinorelbine use (20.0% vs. 62.5%), as presented in Table 3.

**TABLE 3:** Efficacy of Oraxel treatment in all patients.

Parameter	Assessment (*N* = 24)
All response‐evaluable patients, *n* = 23^a^	
ORR, No.(%)	8 (34.8%)
Complete response (CR)	0
Partial response (PR)	8 (34.8%)
Disease control rate (DCR)	21 (91.3%)
Stable disease (SD)	13 (56.5%)
Progressive disease (PD)	2 (8.7%)
ORR, by prior taxane, % (*n*/*N*)	
Prior taxane	25.0% (3/12)
No prior taxane	45.5% (5/11)
ORR, by prior X/G/N, % (n/N)	
Prior X/G/N	20.0% (3/15)
No prior X/G/N	62.5% (5/8)
ITT patients, *n* = 24	
Median Follow up, months (95% CI)	45.71 (42.17–49.24)
Median PFS, months (95% CI)	3.41 (3.15–14.40)
Median PFS with prior taxane, months (95% CI)	
Prior taxane, *n* = 13	3.38 (2.72 to not reached)
No prior taxane, *n* = 11	5.21 (2.56 to not reached)
Median PFS by prior X/G/N, months (95%CI)	
Prior X/G/N, *n* = 15	3.41 (3.15 to not reached)
No prior X/G/N, *n* = 9	5.21 (2.72 to not reached)
Median OS, months (95% CI)	17.90 (14.20 to not reached)
Median OS with prior taxane, months (95% CI)	
Prior taxane, *n* = 13	18.1 (13.8 to not reached)
No prior taxane, *n* = 11	16.9 (11.2 to not reached)
Median OS by prior X/G/N, months (95% CI)	
Prior X/G/N, *n* = 15	14.7 (9.51 to not reached)
No prior X/G/N, *n* = 9	37.9 (16.89 to not reached)

Abbreviations: CI, confidence interval; G, gemcitabine; ITT, intention‐to‐treat; N, vinorelbine; ORR, overall response rate; OS, overall survival; PFS, progression‐free survival; X, capecitabine.

^a^ Subject 002‐S009 was considered to have “no target lesion” at baseline.

**FIGURE 4:**
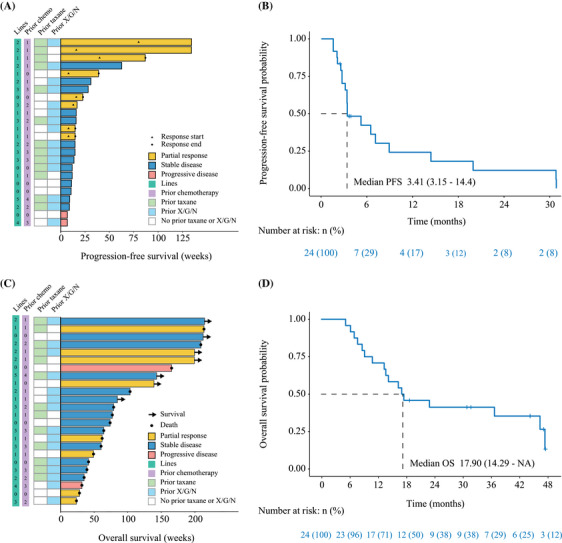
Treatment duration with swimmer plot and Kaplan–Meier curves of progression‐free and overall survival in patients. (A) Swimmer plot with the length of each bar represents the duration treatment for each patient; (B) Kaplan–Meier curves of PFS; (C) swimmer plot with the length of each bar represents the duration survival for each patient; (D) Kaplan–Meier curves of OS. Chemo, chemotherapy; ET, endocrine therapy; OS, overall survival; PFS, progression‐free survival; X/G/N, capecitabine/gemcitabine/ vinorelbine.

As of the data cutoff on June 31, 2023, the median follow‐up time was 45.7 months (95% confidence interval [CI], 42.2–49.2). Figure 4A shows the time to receive treatment for all patients. The median progression‐free survival (PFS) was 3.41 months (95% CI, 3.15–14.40; Figure 4B). Subgroup analyses revealed different PFS outcomes based on prior taxane (3.38 vs. 5.21 months) or capecitabine, gemcitabine, or vinorelbine use (3.41 vs. 5.21 months; Table 3). Figure 4C presents the OS for all patients, with a median OS of 17.90 months (95% CI, 14.29 to not reached; Figure 4D).

In addition, Figure S1 displays the time to receive treatment and OS for four (17%) triple‐negative breast cancer (TNBC) patients, all of whom achieved a disease control rate (DCR). The median PFS for TNBC patients was 8.90 months (95% CI, 2.13 to not reached).

On the other hand, Figure S2 shows the time to receive treatment and OS for 20 (83%) hormone receptor‐positive (HR+) patients, with a median PFS of 3.41 months (95% CI, 3.15–19.9) and a median OS of 17.30 months (95% CI, 11.20 to not reached).

### 2.5 Oraxol significantly alters metabolism and correlates with respond and survival

**FIGURE 5:**
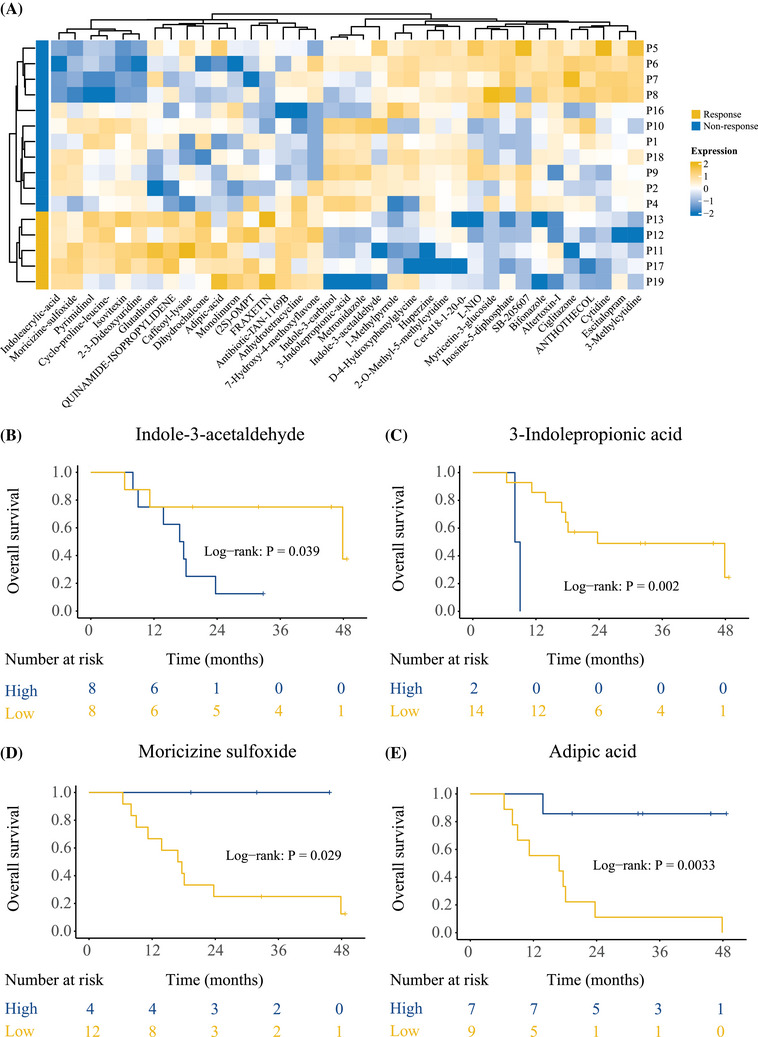
Oraxol significantly alters metabolism correlates with response and overall survival. (A) Differential metabolites with MBC in the PR (response) and SD (no response) groups; (B) indole‐3‐acetaldehyde correlates with OS in MBC patients, *p*<0.05; (C) 3‐indolepropionic acid correlates with OS in MBC patients, *p*<0.05; (D) moricizine sulfoxide correlates with OS in MBC patients, *p*<0.05; and (E) adipic acid correlates with OS in MBC patients, *p*<0.05. MBC, metastatic breast cancer; OS, overall survival; PR, partial response; SD, stable disease.

Metabolic analysis was performed on samples from 16 patients treated with Oraxol, each with varying prognoses, including five patients with PR (response) and 11 patients with SD (no response) (Figure 5A). This analysis identified significant differences in metabolic pathways among the patient groups (Figure 5A, Figure S3), particularly within the tricarboxylic acid (TCA) cycle and pathways related to tryptophan metabolism. Several metabolic pathways were activated in the response group. The tryptophan metabolite indoleacrylic acid, which regulates inflammation and immune metabolism, and another form of indoleacrylic acid involved in amino acid metabolism and protein production, showed high expression. Additionally, metabolites such as glutathione, caffeoyl‐lysine, dihydrochalcone, fraxetin, 7‐hydroxychalcone, and 7‐hydroxy‐4‐methoxyflavone, all associated with antioxidant activity, were also highly expressed. The high levels of glutathione, caffeoyl‐lysine, dihydrochalcone, fraxetin, and 7‐hydroxy‐4‐methoxyflavone suggest that the organism's antioxidant function is highly activated. Furthermore, quinamide‐isopropylidene activity was significantly elevated (Figure 5A). Furthermore, several metabolites of oral paclitaxel were found to influence the efficacy of oncological treatments, particularly those associated with survival outcomes. The differentially expressed metabolites revealed significant alterations in the metabolic pathways of glutathione metabolism, pyrimidine metabolism, and tryptophan metabolism in patients treated with Oraxol. Notably, differential expression of moricizine sulfoxide, adipic acid, indole‐3‐acetaldehyde, and 3‐indolepropionic acid was significantly correlated with OS and PFS in MBC patients (*p* < 0.05, Figure 5B–E, Figure S4).

## 3 DISCUSSION

In this phase I trial, Oraxol demonstrated efficacy in patients with MBC, achieving an ORR of 34.8%, with a median PFS of 3.41 months and a median OS of 17.90 months. In patients with TNBC, the DCR exceeded that of other MBC subtypes, reaching 100%, with a median PFS of 8.90 months. Oraxol demonstrates a unique safety profile, with a high rate of TRAEs but no cases of hypersensitivity reactions or TRAE‐related deaths reported. Additionally, Oraxol significantly alters key metabolic pathways, particularly those associated with the TCA cycle and tryptophan metabolism.

MBC is incurable, and treatment aims to extend survival. Standard guidelines provide distinct therapies for molecular subtypes such as HER2+, HR+, and TNBC.^7^, ^18^, ^19^ Taxanes, particularly paclitaxel, significantly improve OS in treatment failure cases compared to anthracycline‐based treatments.^20^ Moreover, Oraxol remains a viable option for combination therapy in patients who experience disease progression after failing intravenous paclitaxel.^21^ In this study, the ORR for patients previously treated with albuterol was 25%. Therefore, Oraxol paclitaxel may be recommended for future use in patients who have not responded to prior intravenous paclitaxel therapy. Oraxol is expected to gradually replace intravenous paclitaxel in combination with immunotherapy or targeted therapy.

Studies such as IMpassion131 and KEYNOTE‐355 demonstrated improved OS in PD‐L1+ TNBC patients using immune checkpoint inhibitors and nab‐paclitaxel.^22^, ^23^ In this trial, all TNBC patients achieved DCR with a median PFS of 8.9 months, significantly higher than that of other subgroups. Prospectively, TNBC patients may receive Oraxol in combination with immunotherapy to enhance treatment outcomes. In advanced HR+ patients, clinical trials including the APT studies indicated that the combination of paclitaxel with the CDK4/6 inhibitor (palbociclib) or anti‐HER2 inhibitor (trastuzumab) significantly extended PFS.^24^, ^25^ Our study also demonstrates that advanced HR+ MBC patients can still benefit from treatment with Oraxol. On the horizon, Oraxol in combination with CDK4/6 inhibitor, anti‐HER2 inhibitor, and PARP inhibitors therapy is expected to be recommended for patients with HR+ MBC. Additionally, in the CLEOPATRA trial, the median OS was significantly extended to 56.5 months in the group receiving pertuzumab, trastuzumab, and docetaxel in HER2+ breast cancer.^26^ In the PERUSE trial, compared to docetaxel, the median OS of paclitaxel combined with patuximab, trastuzumab therapy exceeded 5 years,^27^ paclitaxel may be a viable replacement for docetaxel as the standard chemotherapy regimen. In the future, Oraxol could be utilized in combination with a HER2 inhibitor or an anti‐HER2 antibody‐drug conjugate for patients with HER2+ breast cancer.

The metabolic pathways differ significantly among patients exhibiting varying efficacy of Oraxol. Metabolites also impact the body's metabolic network, significantly alter the TCA cycle and amino acid metabolic pathways, correlating with OS outcomes and underscoring its potential as an effective treatment option in clinical practice.^28^, ^29^ In patients with response, nucleotide metabolism and tryptophan metabolites are more active, whereas in those with no response, NADPH energy metabolism is more pronounced, suggesting a stronger Warburg effect in patients with modified tumors.^30^, ^31^ The activation of the NAD+ metabolic pathway may lead to immune regulation, nuclear translocation, and DNA repair, thereby promoting tumor growth.^32^ Furthermore, tryptophan‐indole metabolism enhances the metabolic activity of intestinal flora and regulates the growth of intestinal epithelial cells and immune function.^33^ Moreover, nucleotides possess immunostimulatory effects, excessive pyrimidine synthesis is associated with increased sensitivity to immune checkpoint inhibitors,^34^ supporting the potential use of Oraxol in combination with immune checkpoint inhibitors for advanced breast cancer.

Oraxol offers advantages such as convenience, reduced hospital visits, and prolonged drug continuation. Oral administration of chemotherapeutic drugs such as vinorelbine and capecitabine has been shown to be as effective as intravenous administration, and patients prefer this approach to avoid hair loss during treatment.^13^, ^14^ Oral paclitaxel, including DHP107 and Oraxel, shows promise as a convenient and effective treatment for MBC.^15^ DHP107 has been approved for advanced gastric cancer in Korea based on the DREAM study.^13^ Liporaxel demonstrated positive outcomes in a phase II study as a first‐line MBC treatment but lacked later‐line data and had low bioavailability.^16^ This study on Oraxol showed favorable results as an MBC treatment, with a positive response rate and manageable side effects. Furthermore, patients who have previously failed treatment, including those who have received intravenous paclitaxel, may still derive benefit from Oraxol.

Oraxol appears to have a unique safety profile, characterized by a high incidence of TRAE but with no reported hypersensitivity reactions or TRAE‐related fatalities. Notably, only 3% of patients experienced nonfatal treatment‐related SAEs. In contrast, common adverse effects of intravenous spaclitaxel include peripheral neuropathy, primarily presenting as sensory deficits in the limbs, significant myelosuppression, rash, and hypersensitivity reactions.^35^ The incidence of myelosuppression with intravenous paclitaxel was as high as 93.3%. Additionally, hypersensitivity reactions occurred in 30%–41% of patients, with severe reactions in 2%–5% of cases, and 95% of patients experienced these reactions on the first administration.^35^ The incidence of SAEs for intravenous paclitaxel is generally considered higher than that for Oraxol due to the occurrence of hypersensitivity reactions and severe myelosuppression.^36^ Furthermore, SAEs in patients receiving intravenous paclitaxel may include severe allergic reactions, febrile neutropenia, and other serious infections, which can lead to hospitalizations.^37^ The lower incidence of SAEs associated with Oraxol suggests that it may offer a safer alternative for some patients, particularly those at risk for severe allergic reactions linked to intravenous paclitaxel.

This trial underscores the potential of Oraxol therapy in advanced MBC, providing new insights and directions for future research in its management. While indole‐3‐acetaldehyde and 3‐indolepropionic acid have been linked to PFS and OS, they also have the potential to serve as efficacy predictors. However, there are no definitive biomarkers to identify which patients will benefit most from Oraxol therapy currently. Therefore, future studies should focus on uncovering more precise predictors of treatment response. Long non‐coding RNAs, multimodal biomedical, immune cells, and cytokines have the potential to serve as predictive markers of Oraxol efficacy.^38^, ^39^, ^40^ Identifying Oraxol treatment‐related immune cells through single‐cell analyses, along with developing artificial intelligence models that integrate a substantial number of pathological images, computed tomography (CT) images, and magnetic resonance (MR) images from MBC patients,^41^, ^42^ aims to accurately identify populations likely to benefit from Oraxol therapy and predict patient survival times effectively. Artificial intelligence can significantly influence in Phase I and III clinical trials patient recruitment, optimize trial design, and facilitate real‐time data monitoring and analysis.^42^, ^43^ These models are expected to assist clinicians in selecting suitable patients for treatment more effectively, accelerating the development of new therapies and ultimately optimizing treatment outcomes in the future. Prospectively, artificial intelligence may be utilized to aid in trial design, patient enrollment, pharmacological analysis, side effect assessment, efficacy evaluation and comprehensive report in the forthcoming Phase II and III clinical trials of Oraxol.^44^

The study's small sample size limits certainty in Oraxol's effectiveness, requiring validation in a larger cohort. Being a single‐arm study, potential selection bias is possible. Lack of a control group hinders assessing Oraxol's relative efficacy. Further research with proper controls is crucial for robust evidence of Oraxol's therapeutic benefits.

In conclusion, the current study provides valuable insights into the safety, pharmacokinetics, and antitumor activity of Oraxol in advanced breast cancer. Oraxol significantly alters key metabolic pathways, with specific TCA cycle and pathways related to tryptophan metabolisms correlating with respond and survival. The results support the potential use of Oraxol as a treatment option for patients with limited therapeutic alternatives.

## 4 METHODS

### 4.1 Patients

Patients diagnosed with MBC and meeting the specified inclusion and exclusion criteria were deemed eligible to receive the study treatment (Figure 1A). The primary inclusion criteria included the following conditions: (1) Female patients with MBC, aged over 18 years, and an Eastern Cooperative Oncology Group Performance Status (ECOG PS) of 0 or 1. (2) Patients with MBC who had either progressed after prior treatment or were newly diagnosed with MBC. (3) Eligibility is limited to patients with sufficient hematological function, as evidenced by the lack of requirement for transfusion support or granulocyte‐colony stimulating factor (G‐CSF), and who demonstrate stable liver and renal function. (4) Patients expected to survive for no less than 3 months. (5) Eligibility is restricted to patients with quantifiable disease according to the Response Evaluation Criteria in Solid Tumors version 1.1 (RECIST 1.1).

On the other hand, the principal exclusion criteria included the following: (1) Patients who had not fully achieved recovery to ≤ grade 1 toxicity following prior anticancer treatments, including earlier intravenous paclitaxel, as well as from the use of high‐affinity inhibitors (e.g., ketoconazole), stimulators (e.g., rifampin or St. John's Wort) of cytochrome P450 (CYP) 3A4 and CYP2C8, or strong inhibitors or inducers of P‐gp, used within 2 weeks earlier than the initiation of treatment in this study. (2) Patients who received prior treatment with a taxane (paclitaxel or docetaxel) as a component of anthracycline‐inclusive adjuvant chemotherapy or for MBC, and who experienced relapse within 1 year of treatment. (3) Patients with central nervous system metastasis, including leptomeningeal involvement.

These criteria were implemented to ensure that the patient population was suitable for the study and to minimize potential confounding factors that could impact the evaluation of Oraxol's efficacy and safety.

### 4.2 Study design and treatment

Aan open‐label, single‐arm, phase I clinical trial conducted at Sun Yat‐sen Memorial Hospital, Sun Yat‐sen University in China (Figure 1A).

The dosage and administration schedule of the study drugs were as follows: Oraxol, consisting of Oraxol capsules with a dosage of 205 mg/m2 and HM30181A methanesulfonate monohydrate at a concentration of 15 mg, was given once daily for 3 consecutive days each week until disease progression or the onset of unacceptable toxicities (Figure 1B).

During treatment period, patients were admitted to the clinic on the evening prior to Oraxol dosing on week 1, day 1, and they remained at the clinic until the end of pharmacokinetic sampling on day 3. Similarly, patients were readmitted to the clinic the preceding night dosing on week 4, day 1, and remained at the clinic until pharmacokinetic sampling was completed on Day 3 of that week.

### 4.3 Study end points

This Phase I clinical research trial's primary objective is to study the pharmacokinetic parameters of Oraxol in patients with MBC, specifically focusing on the area under the concentration‐time curve (AUC). Secondary endpoints included assessing the safety profile, tolerance, and antineoplastic activity of Oraxol.

For safety, pharmacokinetic, and neoplastic evaluations, CT or magnetic resonance imaging (MRI) imaging was implemented every 8 weeks (at weeks 8 and 16) until cessation of documented progression. These evaluations allowed for monitoring the response to treatment, assessing safety, and determining the pharmacokinetics of Oraxol in patients with MBC.

To evaluate antitumor activity, the ORR was determined based on the proportion of patients in the response evaluable set who achieved a complete response (CR) or PR post‐baseline, as defined by RECIST 1.1. The response assessments were performed by both the investigator and the Independent Central Radiology Review Committee (ICRRC) using CT or MRI. Follow‐up scans are conducted at least 4 weeks later to confirm the patient's objective response.

PFS was characterized as the time from treatment initiation to the first occurrence of either disease progression or death from any cause, whichever occurred first. OS was identified as the time starting with the initiation therapy and going to the death from any cause. Patients were administered Oraxol until disease progression, the development of intolerable toxicity requiring more than two dose reductions, or a maximum of 16 weeks of treatment.

### 4.4 Safety

During the study, AEs and SAEs were evaluated based to the National Cancer Institute Common Terminology Criteria for Adverse Events (NCI‐CTCAE; version 4.03). These evaluations offered critical awareness of the safety and tolerability of Oraxol for MBC.

### 4.5 Pharmacokinetic evaluation

Analytical determination of pharmacokinetic parameters was conducted in patients who provided at least one post‐dose blood sample. The study weeks were counted consecutively starting from week 1 for pharmacokinetic sampling. However, for week 4 pharmacokinetic sampling, a delay was permitted at the investigator's discretion to allow the relevant time to recuperate from any intolerable toxicity, if present. In the instance of treatment postponement, pharmacokinetic samples for week 4 were collected immediately after treatment resumed, regardless of whether a dose reduction were applied. Patients who did not undergo week 4 pharmacokinetic assessments (or corresponding week assessments in case of treatment delay due to toxicity) for any reason were replaced in the analysis.

The pharmacokinetic parameters were synthesized utilizing descriptive data analysis, including mean, standard deviation, median, minimum, and maximum values. What is more, summaries of pharmacokinetic parameters included the geometric average and the coefficient of variation. Individual concentration data and corresponding area under the AUC timepoint data were tabulated in both nominal and actual sampling times for all patients. The concentration‐timepoint data (in actual times) were presented graphically for each subject.

For the analysis, the geometric mean ratio of AUC0‐52, Cmax, Ctrough (24 h), and Ctrough (48 h) in week 4 versus week 1 of treatment was calculated, and two‐sided 90% CIs were determined for comparison. The pharmacokinetic parameters were analyzed using logarithmic transformation, followed by statistical evaluation through analysis of variance, considering subject and treatment effects. Equivalence between the two treatment weeks was concluded if the 90% CIs of AUC0‐52, Cmax, Ctrough (24 h), and Ctrough (48 h) fell within the range of 80%–125%. This analysis allowed for the assessment of the pharmacokinetic profile of Oraxol over the course of treatment.

### 4.6 Trial oversight

The trial is recorded on ClinicalTrials.gov under the identifier NCT04993040. Ethical approval and informed consent were obtained, following Good Clinical Practice guidelines and the Declaration of Helsinki. Signed informed consent was obtained from each participant.

### 4.7 Blood sample collection and preparation

Patient blood samples were designated as P1, P2, P3… P15, P16. To 200 µL of peripheral blood plasma samples, about 800 µL methanol was included, followed by shaking for 30 s and ultrasound treatment for 10 min. Subsequently, the mixtures were maintained at −20°C about 2 h and followed by centrifugation at 13,000 rpm at 4°C for 15 min to collect the supernatants. Following this, vacuum drying was performed, and the desiccated residues were re‐solubilized in methanol/water (1:1, v/v) about 200 µL, and the resulting products were analyzed for metabolomics assessment using ultra‐high‐performance liquid chromatography and mass spectrometry.

Sample profiling was conducted using the Ekspert UltraLC 110 (AB Sciex, Framingham) coupled with the Triple TOF 5600+ (AB Sciex, Framingham). For chromatographic separation, an ACQUITY UPLC HSS T3 column (2.1 mm × 100 mm, 1.8 µm, Waters) was employed, and the column temperature was maintained at 35°C. Note that 1:9 acetonitrile/water (v/v) solution contained 0.1% formic acid for mobile phase A, while 9:1 acetonitrile/water (v/v) solution containing 0.1% formic acid was used to form mobile phase B. The mass spectrometry parameters were as follows: ion spray voltage of 5500 V, setting curtain gas pressure at 40 Pa, starting temperature of 550°C, collision‐induced dissociation energies set at 30 eV, MS1 scan range from 50 to 1000 *m*/*z*, and MS2 scan range from 25 to 1000 *m*/*z*.

### 4.8 Metabolites identification and analysis

All products were identified by comparing them with entries in the MassBank of North America (http://mona.fiehnlab.ucdavis.edu/) and the HMDB database (http://www.hmdb.ca). Metabolite identification was considered valid if the mass discrepancy comparing the observed value and the database value did not exceed 0.025 Da.

The pattern recognition techniques implemented in MetaboAnalyst 3.0 are employed for the analysis of complex datasets. To mitigate concentration disparities among samples, the data were standardized to the cumulative spectral peak intensity, and logarithmic normalization were employed in nonlinear correction for normalization of skewed distributions. Automatic scaling was employed to ensure comparability among variables. For one‐variable statistical analysis, the meaningful statistical difference of characteristics across the PR samples and SD samples were determined utilizing MetaboAnalyst 3.0 for *t*‐test analysis, with a significance threshold of *p* < 0.05. MetaboAnalyst 3.0 was used to conduct metabolomic pathway analysis to find the metabolic pathways most relevant to PR.

### 4.9 Statistical analysis

PFS and OS were evaluated through the Kaplan–Meier method, with 95% CIs calculated to provide an estimation of the reliability of the survival probabilities. The log‐rank test was employed to compare survival curves between groups, with statistical significance defined as a *p*‐value < 0.05.

The ORR was calculated as the proportion of patients achieving a complete or PR, while the incidence rates of AEs and SAEs were computed based on the total number of treated patients, expressed as percentages. These were further categorized by severity and relationship to treatment.

All statistical analyses were performed using R software (version 4.2.1), utilizing appropriate statistical packages for survival analysis, hypothesis testing and exploratory data analysis. A significance threshold of *p* < 0.05 was applied for all tests, and results were interpreted with respect to clinical relevance in addition to statistical significance. In R, visualizations were created with ComplexHeatmap, ggplot2, and ggpubr.

## AUTHOR CONTRIBUTIONS

*Concept and design*: Heuri Yao, Tao Qin, Ying Wang, and Junyan Wu. *Acquisition*: All authors. *Analysis or interpretation of data*: Tao Qin, Ying Wang, Suiwen Ye, Yunfang Yu, and Luhui Mao. *Drafting of the manuscript*: All authors. *Critical revision of the manuscript for important intellectual content*: All authors. *Statistical analysis*: Tao Qin, Ying Wang, Suiwen Ye, Yunfang Yu, and Luhui Mao. *Obtained funding*: Herui Yao. *Administrative, technical, or material support*: All authors. *Supervision*: Herui Yao. All authors have full access to all the data in the study, take responsibility for integrity of the data and the accuracy of the data analysis, and have read and approved the final manuscript.

## CONFLICT OF INTEREST STATEMENT

The authors declare no conflicts of interest.

## ETHICS STATEMENT

Ethical approval and informed consent were obtained, following Good Clinical Practice guidelines and the Declaration of Helsinki. Each participant provided written informed consent. This clinical study was approved by the Medical Ethics Committee of Sun Yat‐sen Memorial Hospital, Sun Yat‐sen University, under the approval number [2018] Lun Shen Yao No. (82), with clinical trial registration No. NCT04993040.

## Supporting information



Supporting Information

## Data Availability

The data are governed by data usage policies specified by the data controller (Sun Yat‐sen Memorial Hospital, Sun Yat‐sen University). To protect the privacy of the patients, all data will be available for non‐commercial research purposes upon approval by the corresponding author Herui Yao for 10 years upon reasonable request approved by the Ethics Committee.

## References

[1] Kalinsky K , Barlow WE , Gralow JR , et al. 21‐Gene assay to inform chemotherapy benefit in node‐positive breast cancer. N Engl J Med. 2021;385(25):2336‐2347. 34914339 10.1056/NEJMoa2108873 PMC9096864

[2] Schmid P , Cortes J , Dent R , et al. KEYNOTE‐522 investigators. event‐free survival with pembrolizumab in early triple‐negative breast cancer. N Engl J Med. 2022;386(6):556‐567. 35139274 10.1056/NEJMoa2112651

[3] Agostinetto E , Gligorov J , Piccart M , Systemic therapy for early‐stage breast cancer: learning from the past to build the future. Nat Rev Clin Oncol. 2022;19(12):763‐774. 36253451 10.1038/s41571-022-00687-1 PMC9575647

[4] Sung H , Ferlay J , Siegel RL , et al. Global cancer statistics 2020: GLOBOCAN estimates of incidence and mortality worldwide for 36 cancers in 185 countries. CA Cancer J Clin. 2021;71(3):209‐249. 33538338 10.3322/caac.21660

[5] Pe M , Dorme L , Coens C , et al. Statistical analysis of patient‐reported outcome data in randomised controlled trials of locally advanced and metastatic breast cancer: a systematic review. Lancet Oncol. 2018;19(9): e459‐e469. 30191850 10.1016/S1470-2045(18)30418-2

[6] Hijazi A , Mohanna M , Sabbagh S , et al. Clinico‐pathologic factors and survival of patients with breast cancer diagnosed with de novo brain metastasis: a national cancer database analysis. Breast Cancer Res Treat. 2024;206(3):527‐541. 38683296 10.1007/s10549-024-07321-x PMC11208224

[7] Gradishar WJ , Moran MS , Abraham J , et al. Breast cancer, version 3.2022, NCCN clinical practice guidelines in oncology. J Natl Compr Canc Netw. 2022;20(6):691‐722. 35714673 10.6004/jnccn.2022.0030

[8] Waks AG , Winer EP , Breast cancer treatment: a review. JAMA. 2019;321(3):288‐300. 30667505 10.1001/jama.2018.19323

[9] Bonotto M , Gerratana L , Poletto E , et al. Measures of outcome in metastatic breast cancer: insights from a real‐world scenario. Oncologist. 2014;19(6):608‐615. 24794159 10.1634/theoncologist.2014-0002 PMC4041678

[10] Joensuu H , Kellokumpu‐Lehtinen PL , Huovinen R , et al. Adjuvant capecitabine for early breast cancer: 15‐year overall survival results from a randomized trial. J Clin Oncol. 2022;40(10):1051‐1058. 35020465 10.1200/JCO.21.02054 PMC8966968

[11] Mittendorf EA , Zhang H , Barrios CH , et al. Neoadjuvant atezolizumab in combination with sequential nab‐paclitaxel and anthracycline‐based chemotherapy versus placebo and chemotherapy in patients with early‐stage triple‐negative breast cancer (IMpassion031): a randomised, double‐blind, phase 3 trial. Lancet. 2020;396(10257):1090‐1100. 32966830 10.1016/S0140-6736(20)31953-X

[12] Schmid P , Adams S , Rugo HS , et al. Atezolizumab and nab‐paclitaxel in advanced triple‐negative breast cancer. N Engl J Med. 2018;379(22):2108‐2121. 30345906 10.1056/NEJMoa1809615

[13] Kang YK , Ryu MH , Park SH , et al. Efficacy and safety findings from DREAM: a phase III study of DHP107 (Oraxol) versus i.v. paclitaxel in patients with advanced gastric cancer after failure of first‐line chemotherapy. Ann Oncol. 2018;29(5):1220‐1226. 29438463 10.1093/annonc/mdy055

[14] Kim SB , Seo JH , Ahn JH , et al. Phase II study of DHP107 (Oraxol) in the first‐line treatment of HER2‐negative recurrent or metastatic breast cancer (OPTIMAL study). Ther Adv Med Oncol. 2021;13:17588359211061989. 34925553 10.1177/17588359211061989 PMC8679020

[15] Jang Y , Chung HJ , Hong JW , et al. Absorption mechanism of DHP107, an Oraxol formulation that forms a hydrated lipidic sponge phase. Acta Pharmacol Sin. 2017;38(1):133‐145. 27867185 10.1038/aps.2016.105 PMC5220538

[16] Kim TE , Lee H , Lim KS , et al. Effects of HM30181, a P‐glycoprotein inhibitor, on the pharmacokinetics and pharmacodynamics of loperamide in healthy volunteers. Br J Clin Pharmacol. 2014;78(3):556‐564. 24602137 10.1111/bcp.12368 PMC4243906

[17] Huang L , Wang X , Zhou L , et al. Oral vinorelbine versus intravenous vinorelbine, in combination with epirubicin as first‐line chemotherapy in Chinese patients with metastatic breast cancer. Cancer Chemother Pharmacol. 2020;85(1):205‐215. 31838561 10.1007/s00280-019-04000-3 PMC6994442

[18] Cardoso F , Paluch‐Shimon S , Senkus E , et al. 5th ESO‐ESMO international consensus guidelines for advanced breast cancer (ABC 5). Ann Oncol. 2020;31(12):1623‐1649. 32979513 10.1016/j.annonc.2020.09.010 PMC7510449

[19] Burstein HJ , Somerfield MR , Barton DL , et al. Endocrine treatment and targeted therapy for hormone receptor‐positive, human epidermal growth factor receptor 2‐negative metastatic breast cancer: ASCO guideline update. J Clin Oncol. 2021;39(35):3959‐3977. 34324367 10.1200/JCO.21.01392 PMC8659999

[20] Ghersi D , Willson ML , Chan MM , et al. Taxane‐containing regimens for metastatic breast cancer. Cochrane Database Syst Rev. 2015;2015(6):CD003366. 26058962 10.1002/14651858.CD003366.pub3 PMC6464903

[21] Bishop JF , Dewar J , Toner GC , et al. Initial paclitaxel improves outcome compared with CMFP combination chemotherapy as front‐line therapy in untreated metastatic breast cancer. J Clin Oncol. 1999;17(8):2355‐2364. 10561297 10.1200/JCO.1999.17.8.2355

[22] Miles D , Gligorov J , André F , et al. Primary results from IMpassion131, a double‐blind, placebo‐controlled, randomised phase III trial of first‐line paclitaxel with or without atezolizumab for unresectable locally advanced/metastatic triple‐negative breast cancer. Ann Oncol. 2021;32(8):994‐1004. 34219000 10.1016/j.annonc.2021.05.801

[23] Cortes J , Rugo HS , Cescon DW , et al. Pembrolizumab plus chemotherapy in advanced triple‐negative breast cancer. N Engl J Med. 2022;387(3):217‐226. 35857659 10.1056/NEJMoa2202809

[24] Huppert LA , Gumusay O , Idossa D , et al. Systemic therapy for hormone receptor‐positive/human epidermal growth factor receptor 2‐negative early stage and metastatic breast cancer. CA Cancer J Clin. 2023;73(5):480‐515. 36939293 10.3322/caac.21777

[25] Tolaney SM , Tarantino P , Graham N , et al. Adjuvant paclitaxel and trastuzumab for node‐negative, HER2‐positive breast cancer: final 10‐year analysis of the open‐label, single‐arm, phase 2 APT trial. Lancet Oncol. 2023;24(3):273‐285. 36858723 10.1016/S1470-2045(23)00051-7

[26] Swain SM , Miles D , Kim SB , et al. Pertuzumab, trastuzumab, and docetaxel for HER2‐positive metastatic breast cancer (CLEOPATRA): end‐of‐study results from a double‐blind, randomised, placebo‐controlled, phase 3 study. Lancet Oncol. 2020;21(4):519‐530. 32171426 10.1016/S1470-2045(19)30863-0

[27] Miles D , Ciruelos E , Schneeweiss A , et al. Final results from the PERUSE study of first‐line pertuzumab plus trastuzumab plus a taxane for HER2‐positive locally recurrent or metastatic breast cancer, with a multivariable approach to guide prognostication. Ann Oncol. 2021;32(10):1245‐1255. 34224826 10.1016/j.annonc.2021.06.024

[28] De Martino M , Rathmell JC , Galluzzi L , et al. Cancer cell metabolism and antitumour immunity. Nat Rev Immunol. 2024;24(7): 537. 38806693 10.1038/s41577-024-01051-3

[29] Sivanand S , Vander Heiden MG . Emerging roles for branched‐chain amino acid metabolism in cancer. Cancer Cell. 2020;37(2):147‐156. 32049045 10.1016/j.ccell.2019.12.011 PMC7082774

[30] Icard P , Shulman S , Farhat D , et al. How the Warburg effect supports aggressiveness and drug resistance of cancer cells? Drug Resist Updat. 2018;38:1‐11. 29857814 10.1016/j.drup.2018.03.001

[31] Schwartz L , Seyfried T , Alfarouk KO , et al. Out of Warburg effect: an effective cancer treatment targeting the tumor specific metabolism and dysregulated pH. Semin Cancer Biol. 2017;43:134‐138. 28122260 10.1016/j.semcancer.2017.01.005

[32] Ma C , Zheng K , Jiang K , et al. The alternative activity of nuclear PHGDH contributes to tumour growth under nutrient stress. Nat Metab. 2021;3(10):1357‐1371. 34663976 10.1038/s42255-021-00456-x

[33] Xue C , Li G , Zheng Q , et al. Tryptophan metabolism in health and disease. Cell Metab. 2023;35(8):1304‐1326. 37352864 10.1016/j.cmet.2023.06.004

[34] Bader JE , Voss K , Rathmell JC . Targeting metabolism to improve the tumor microenvironment for cancer immunotherapy. Mol Cell. 2020;78(6):1019‐1033. 32559423 10.1016/j.molcel.2020.05.034 PMC7339967

[35] Gradishar WJ , Tjulandin S , Davidson N , et al. Phase III trial of nanoparticle albumin‐bound paclitaxel compared with polyethylated castor oil‐based paclitaxel in women with breast cancer. J Clin Oncol. 2005;23(31):7794‐803. 16172456 10.1200/JCO.2005.04.937

[36] Rugo HS , Umanzor GA , Barrios FJ , et al. Open‐label, randomized, multicenter, phase III study comparing oral paclitaxel plus encequidar versus intravenous paclitaxel in patients with metastatic breast cancer. J Clin Oncol. 2022;20:JCO2102953. 10.1200/JCO.21.02953 PMC9788977 35858154

[37] Lang I , Inbar MJ , Kahán Z , et al. Safety results from a phase III study (TURANDOT trial by CECOG) of first‐line bevacizumab in combination with capecitabine or paclitaxel for HER‐2‐negative locally recurrent or metastatic breast cancer. Eur J Cancer. 2012;48(17):3140‐9. 22640829 10.1016/j.ejca.2012.04.022

[38] Yu Y , Chen H , Ouyang W , et al. Unraveling the role of M1 macrophage and CXCL9 in predicting immune checkpoint inhibitor efficacy through multicohort analysis and single‐cell RNA sequencing. MedComm, 2024;5(3):e471. 38434763 10.1002/mco2.471 PMC10906808

[39] Yu Y , Zhang W , Li A , et al. Association of long non‐coding RNA biomarkers with clinical immune subtype and prediction of immunotherapy response in patients with cancer. JAMA Network Open. 2020;3(4):e202149. 32259264 10.1001/jamanetworkopen.2020.2149 PMC7139278

[40] Acosta JN , Falcone GJ , Rajpurkar P , et al. Multimodal biomedical AI. Nat Med. 2022;28(9):1773‐1784. 36109635 10.1038/s41591-022-01981-2

[41] Wang D , Feng L , Ye J , et al. Accelerating the integration of ChatGPT and other large‐scale AI models into biomedical research and healthcare. MedComm–Future Med 2023;2:e43.

[42] Cao P‐W , Deng X‐Y , Pan Y‐P , et al. Early prediction of neoadjuvant chemotherapy efficacy for mass breast cancer based on dynamic contrast‐enhanced magnetic resonance imaging radiomics. MedComm–Oncology 2024;3:e84

[43] Hutson M . How AI is being used to accelerate clinical trials. Nature. 2024;627(8003):S2‐S5. 38480968 10.1038/d41586-024-00753-x

[44] Aliper A , Kudrin R , Polykovskiy D , et al. Prediction of clinical trials outcomes based on target choice and clinical trial design with multi‐modal artificial intelligence. Clin Pharmacol Ther. 2023;114(5):972‐980. 37483175 10.1002/cpt.3008

