# Supplementary material for: Phase I clinical trial to assess safety and efficacy of Oraxol, a novel oral paclitaxel chemotherapy agent, in patients with previously treated metastatic breast cancer
Source: MedComm (2020). 2025 Feb 17;6(3):e70097. doi: 10.1002/mco2.70097 (PMC11831190; doi:10.1002/mco2.70097)
Supplement: Supplementary file 1 — Supporting Information [file MCO2-6-e70097-s001.docx]

**Supplementary Online Content**

**Figure S1.** **Treatment Duration With Swimmer Plot and Kaplan-Meier Curves of Progression-free Survival in Triple-negative Breast Cancer Patients.**

**Figure S2. Treatment Duration With Swimmer Plot and Kaplan-Meier Curves of Progression-free Survival and Overall Survival in ER(+)/PR(+) patients.**

**Figure S3.** **The Differentially Expressed Metabolites Between the Response and Non-response in Metastatic Breast Cancer patients.**

**Figure S4.** **Oraxol Significantly Alters Metabolism Correlates With PFS in Metastatic Breast Cancer Patients.**

**Table S1.** **Pharmacokinetic Parameters for Oraxel.**

**Table S2. Grade 3 or Higher TEAE.**

**Figure S1.** **Treatment Duration With Swimmer Plot and Kaplan-Meier Curves of Progression-free Survival in Triple-negative Breast Cancer Patients.**

**
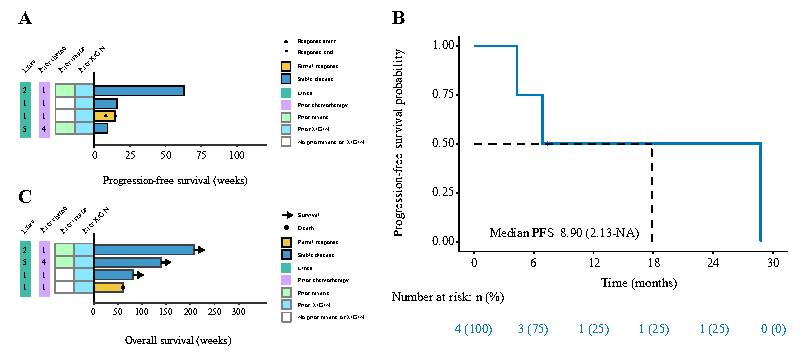
**

(A) Swimmer plot with the length of each bar represents the duration treatment for each triple-negative breast cancer patient; (B) Kaplan-Meier curves of PFS in triple-negative breast cancer patients; (C) Swimmer plot with the length of each bar represents the duration survival for each triple-negative breast cancer patient. Chemo, chemotherapy; ET, endocrine therapy; X/G/N, capecitabine/gemcitabine/ vinorelbine; PFS, progression-free survival; OS, overall survival.

**Figure S2.** **Treatment Duration With Swimmer Plot and Kaplan-Meier Curves of Progression-free Survival and Overall Survival in ER(+)/PR(+) patients.**

**
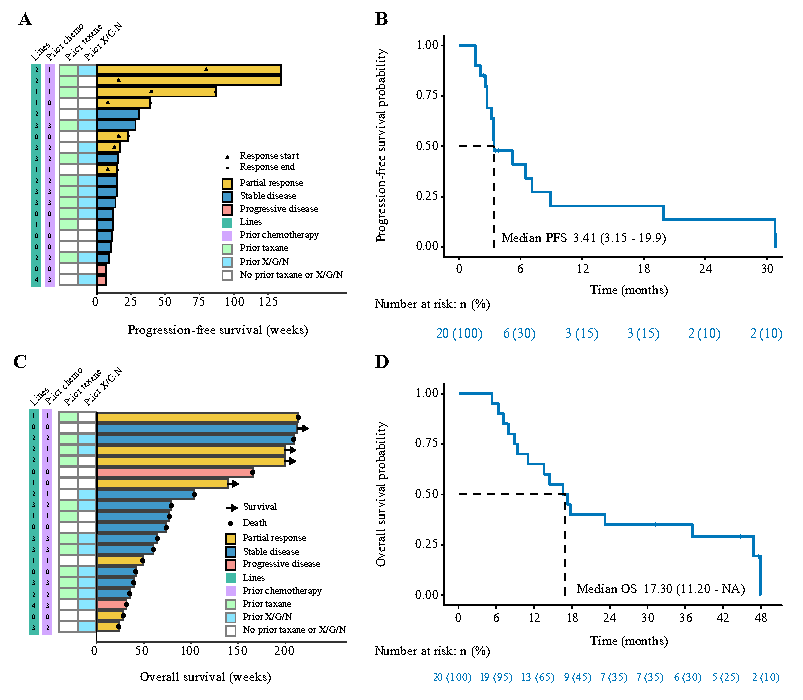
**

(A) Swimmer plot with the length of each bar represents the duration treatment for each ER(+)/PR(+) patient; (B) Kaplan-Meier curves of PFS in ER(+)/PR(+) patients; (C) Swimmer plot with the length of each bar represents the duration survival for each ER(+)/PR(+) patient; (D) Kaplan-Meier curves of OS in ER(+)/PR(+) patients. Chemo, chemotherapy; ET, endocrine therapy; X/G/N, capecitabine/gemcitabine/vinorelbine; ER, estrogen receptor; PR, progesterone receptor; PFS, progression-free survival; OS, overall survival.

**Figure S3. The Differentially Expressed Metabolites Between the Response and Non-response in Metastatic Breast Cancer Patients.**

**
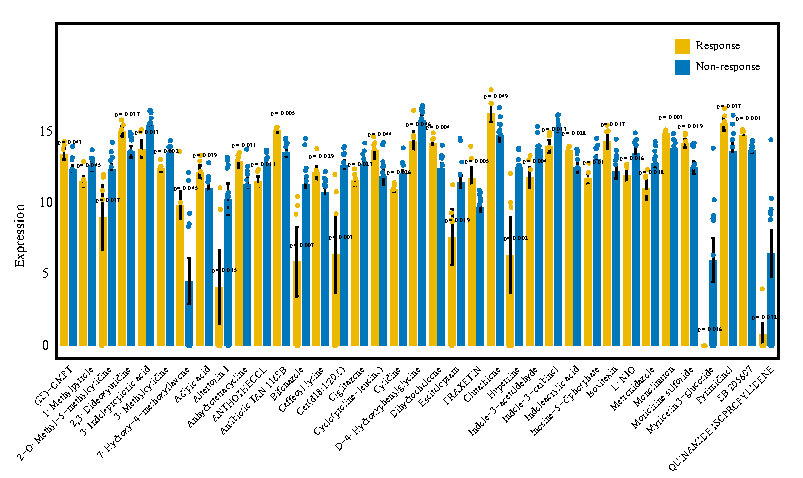
**

**Figure S4. Oraxol Significantly Alters Metabolism Correlates With PFS in Metastatic Breast Cancer patients.**

**
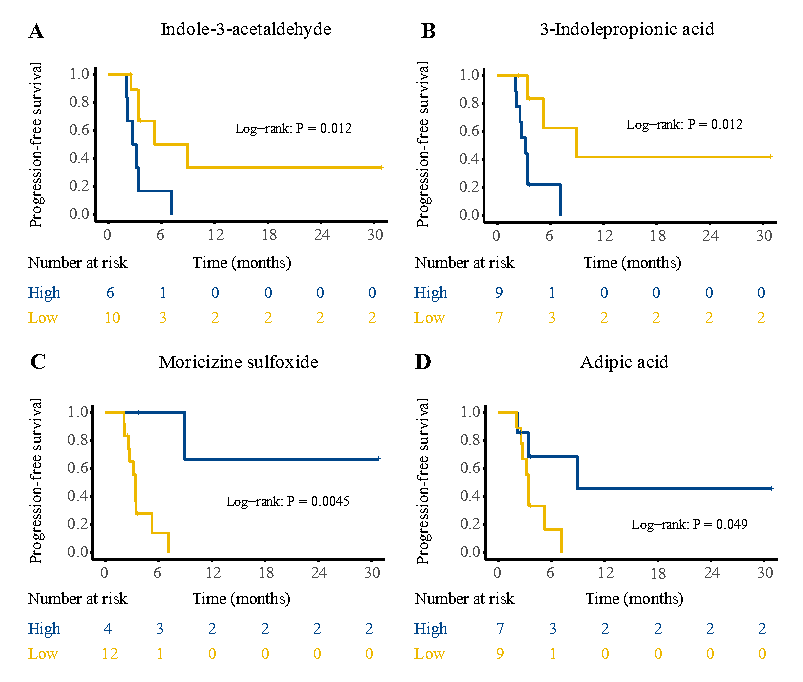
**

(A) Indole-3-acetaldehyde correlates with PFS in MBC patients, *P*＜0.05; (C) 3-Indolepropionic acid correlates with PFS in MBC patients, *P*＜0.05; (D) Dihydrochalcone correlates with PFS in MBC patients, *P*＜0.05; (E) Adipic acid correlates with PFS in MBC patients, *P*＜0.05. PFS, progression-free survival; MBC, metastatic breast cancer.

**Table S1. Pharmacokinetic Parameters for Oraxel.**

|  | **AUC (0-52)** | | **Cmax** | | **Cmax (0-24)** | | **Cmax ­(24-48)** | |
| --- | --- | --- | --- | --- | --- | --- | --- | --- |
| Days | Mean | % | Mean | % | Mean | % | Mean | % |
| Paclitaxel |  |  |  |  |  |  |  |  |
| Study day 1 | 3544.6 | 41.4 | 403.9 | 45.2 | 288.5 | 53.8 | 337.6 | 51.9 |
| Study day 22 | 3524.2 | 37.8 | 398.5 | 45.4 | 328.8 | 55.1 | 289.3 | 44.7 |
| HM30181A |  |  |  |  |  |  |  |  |
| Study day 1 | 74.5 | 47.34 | 1.35 | 49.3 | 1.35 | 49.3 | 2.32 | 43.73 |
| Study day 22 | 88.3 | 44.4 | 1.67 | 43.8 | 1.67 | 43.8 | 2.5 | 40.48 |
|  | **Cmax (48-52)** | | **Tmax (0-24)** | | **Tmax (24-48)** | | **Tmax (48-52)** | |
|  | **Mean** | **%** | **Median** | **Range** | **Median** | **Range** | **Median** | **Range** |
| Paclitaxel |  |  |  |  |  |  |  |  |
| Study day 1 | 324.5 | 53.4 | 1.0 | 1.0 to 2.0 | 25.5 | 25.0 to 27 | 49.3 | 49.0 to 51.0 |
| Study day 22 | 312.7 | 59.2 | 1.0 | 1.0 to 2.0 | 25.0 | 25.0 to 27 | 49.0 | 49.0 to 51.1 |
| HM30181A |  |  |  |  |  |  |  |  |
| Study day 1 | 2.9 | 49.67 | 5.0 | 4.0 to 24.9 | 29.0 | 27.0 to 29.0 | 53.0 | 52.0 to 53.0 |
| Study day 22 | 3.02 | 37.78 | 4.5 | 2.0 to 24.8 | 29.0 | 26.0 to 29.1 | 53.0 | 50.0 to 53.1 |

| Table S2. Grade 3 or Higher TEAE. | |
| --- | --- |
| Preferred term, n (%) | **All Patients (N = 24)** |
| **Number (%) of patients with at least grade 3 or higher TEAE** | **15 (63%)** |
| Grade 3 TEAE | 11 (46%) |
| Grade 4 TEAE | 4 (17%) |
| Grade 5 TEAE | 0 |
| Decreased white blood cell count | 8 (33%) |
| Decreased neutrophil count | 11 (46%) |
| **Vascular disorders** | **3 (13%)** |
| Hypertension | 2 (8%) |
| **Blood and lymphatic system disorders** | **2 (8%)** |
| Abbreviation: TEAE, treatment‑emergent adverse event. | |
